# Interscalene brachial plexus nerve block in the emergency department: an effective and practice-changing workshop

**DOI:** 10.1186/s13089-019-0131-x

**Published:** 2019-07-15

**Authors:** Tyler Beals, Kay Odashima, Lawrence E. Haines, Antonios Likourezos, Jefferson Drapkin, Eitan Dickman

**Affiliations:** 1 Beth Israel Deaconess Medical Center, 1 Deaconess Rd, Rosenberg 2, Boston, MA 02215 USA; 2 Maimonides Medical Center, Brooklyn, NY USA

**Keywords:** Emergency medicine, Point of care ultrasound, Regional anesthesia, Interscalene brachial plexus nerve block, Shoulder dislocation, Pain management, Medical education

## Abstract

**Background:**

The interscalene brachial plexus nerve block (ISNB) is a potentially useful method of regional analgesia for humerus fracture and shoulder dislocation reduction in the Emergency Department (ED). We examined the effectiveness of an ISNB workshop given to emergency medicine (EM) residents. We also explored complication rates and effectiveness of ISNBs performed in the ED.

**Methods:**

One-hour evidence-based ISNB workshops were conducted with EM residents. Participants were given pre-, post-, and 3-month post-workshop knowledge and technical assessments. Results were analyzed using descriptive statistics. A pre- and post-workshop chart review examined ISNB utilization, complications, post-ISNB opiate administration, and post-ISNB procedural sedation.

**Results:**

41 residents enrolled in the workshop. Pre-workshop pass rate: knowledge assessment 22%. Immediate post-workshop pass rates: knowledge assessment 100%, image acquisition 93%, needle placement 100%. Three months post-workshop pass rates: knowledge assessment 73%, image acquisition 76%, needle placement 100%. Areas of poorest knowledge retention were anatomical landmarks, block distribution, and early signs of LAST. In the chart review, 2 ISNBs were performed in the pre-workshop period, and 12 in the post-workshop period. No serious complications were recorded. 78.5% of attempted ISNBs were successful, without need for procedural sedation. Of the 11 successfully performed ISNBs, 91% received no opiates after the procedure.

**Conclusions:**

Our study suggests that EM residents can learn the ISNB, perform it safely in the emergency department, and that the ISNB may be an alternative to procedural sedation and opiate use for shoulder dislocation. Residents are adept at ISNB technical skills but demonstrate some deficits in knowledge retention.

## Background

Ultrasound-guided regional anesthesia (UGRA) has been used in peri-operative settings by anesthesiologists for decades and is now becoming increasingly common in Emergency Departments (EDs) [4]. Particularly in the setting of the current opioid crisis, there is growing interest in opioid-sparing analgesic techniques [3, 7, 21, 23]. A recent AAEM position paper on the management of acute pain in the ED calls for “pain-syndrome targeted” strategies including regional anesthesia [13].

The interscalene brachial plexus nerve block (ISNB) is well established in anesthesia and orthopedic literature to provide effective analgesia for shoulder surgery and humerus fractures [1, 12, 18]. A small body of literature supports the usefulness of brachial plexus nerve blocks for ED-relevant indications including humerus fracture, shoulder reduction, and deltoid abscess drainage [5, 17, 20, 22]. In addition to analgesia, regional anesthesia can decrease length of stay (LOS) through avoidance of procedural sedation [5, 17, 20, 22].

Bedside ultrasound training is the standard in American emergency medicine (EM) residencies, and EPs are facile in its various applications. The 2013 Council of Emergency Medicine Residency Directors-Academy of Emergency Ultrasound (CORD-AEUS) consensus document recommends UGRA, and specifically the ISNB, as advanced skills to be taught in emergency medicine residency [9].

However, there is currently a paucity of literature describing effective educational techniques for EM residents in UGRA. Until recently, UGRA education was largely limited to descriptions in the anesthesia literature, and even there, universal agreement on the most effective methods does not exist [16]. Akthar et al. demonstrated femoral nerve block competency in EM residents after a brief workshop [2]. This workshop was 1-h long and included didactic and simulator training. Bretholz et al. showed increased self-reported confidence in pediatric EM residents’ performance of ulnar and femoral nerve blocks after a half-day workshop but did not assess competency [6]. Other literature suggests that use of simulators and dedicated workshops are effective methods of teaching UGRA [10, 11, 15, 19]. Regarding the ISNB specifically, EPs have been found to be capable of identifying the relevant anatomy and needle path after a brief educational course [8].

The goal of this study is to assess the feasibility of implementing a 1-h workshop teaching the ISNB to EM residents. A secondary aim is a retrospective examination of the clinical utilization, safety, and efficacy of ISNBs performed in the ED before and after the workshop.

## Materials and methods

This study was approved by Maimonides Medical Center Institutional Review Board. All participants in the workshop provided written informed consent to participate in this study. The training sessions took place at a large urban tertiary academic medical center with an active emergency ultrasound program, including fellowship training. The workshop design was adapted from a previous study of the ultrasound-guided femoral nerve block [2] as well as other evidence-based educational methods described in the literature. Educational goals of the workshop, as described in the sections below, were based upon UGRA educational guidelines set forth by the American Society of Regional Anesthesia and Pain Medicine and the European Society of Regional Anaesthesia and Pain Therapy Joint Committee [14]. The 1-h workshop was taught by an emergency ultrasound fellowship trained physician and a senior EM resident. Multiple workshop dates were held over the course of 6 months with groups of 2–4 residents in their 1st, 2nd, or 3rd year of training. Residents involved had prior training in core emergency ultrasound applications and US-guided needle placement for vascular access as part of their residency training.

The workshop consisted of four sections:

1. General knowledge and safety: This section consisted of a brief lecture covering the indications for an ISNB, its distribution of analgesia, appropriate dose and type of anesthetic, aseptic technique, visualization of the needle and anesthetic spread, avoidance of complications, and management of local anesthetic systemic toxicity. A video demonstration of the procedure was shown.
2. Image recognition: Residents were shown a series of ultrasound images of the interscalene brachial plexus and its surrounding anatomy. They were taught relevant sonographic landmarks and appropriate needle trajectories for performing a successful block.
3. Image acquisition: Residents identified sonographic landmarks and located the interscalene brachial plexus on themselves and each other.
4. Motor skill: Finally, they practiced in-plane needle placement on an ISNB simulation model.

Pre-, immediate post-, and 3 months post-workshop assessments were conducted to examine the effectiveness of the workshop and skill retention. The assessment consisted of three parts:

1. Paper assessment: This was a 10-item multiple choice exam covering general knowledge of UGRA and the ISNB in particular (Fig. 1). A score of 80% was considered passing.

**Fig. 1:**
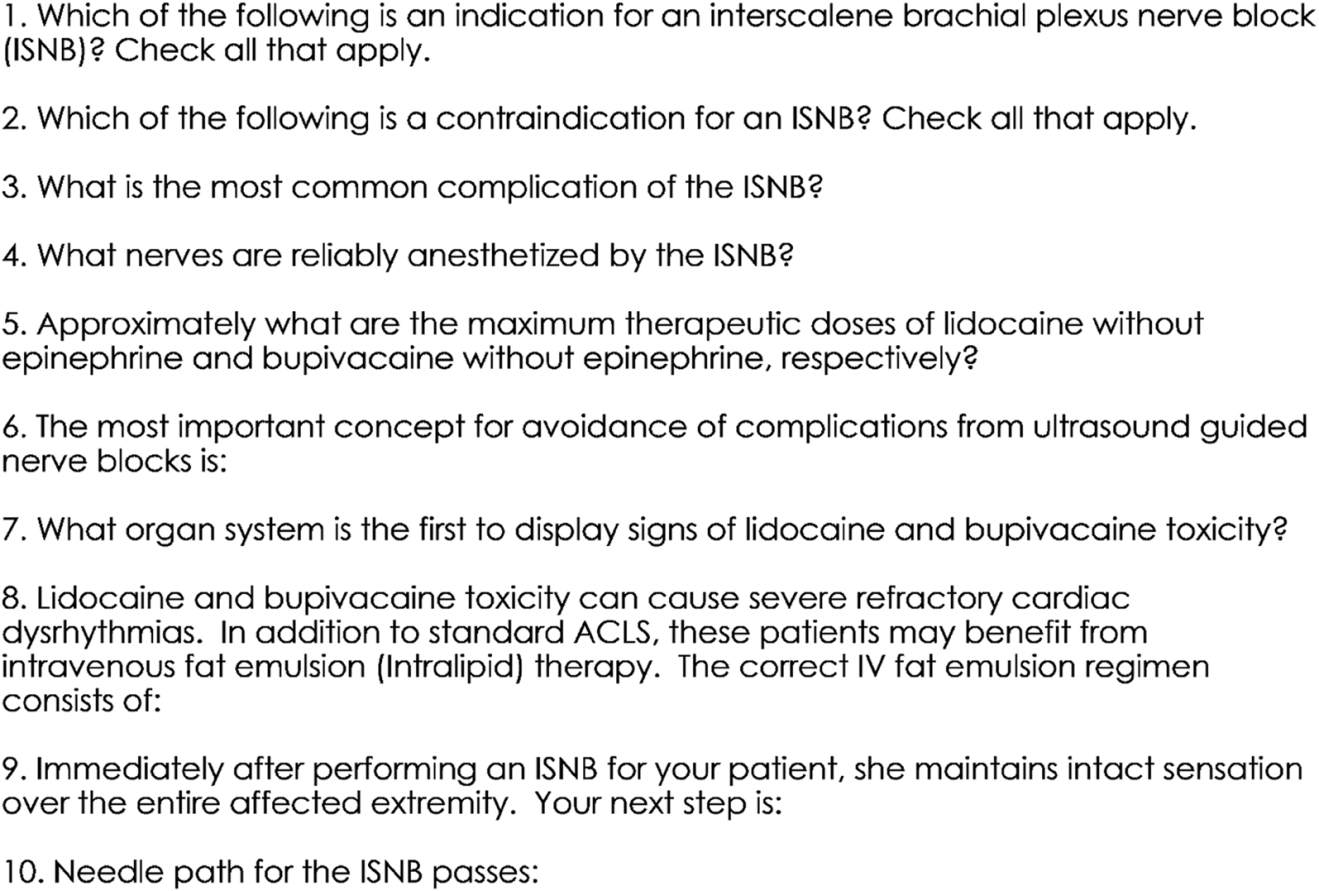
Paper assessment. Multiple choice answers not shown
2. Image acquisition assessment: Using healthy volunteers, residents identified the sternocleidomastoid, anterior scalene, middle scalene, brachial plexus roots, carotid artery, injection site, and planned needle path (Fig. 2). Correct identification of all of this was required for passage.

**Fig. 2:**
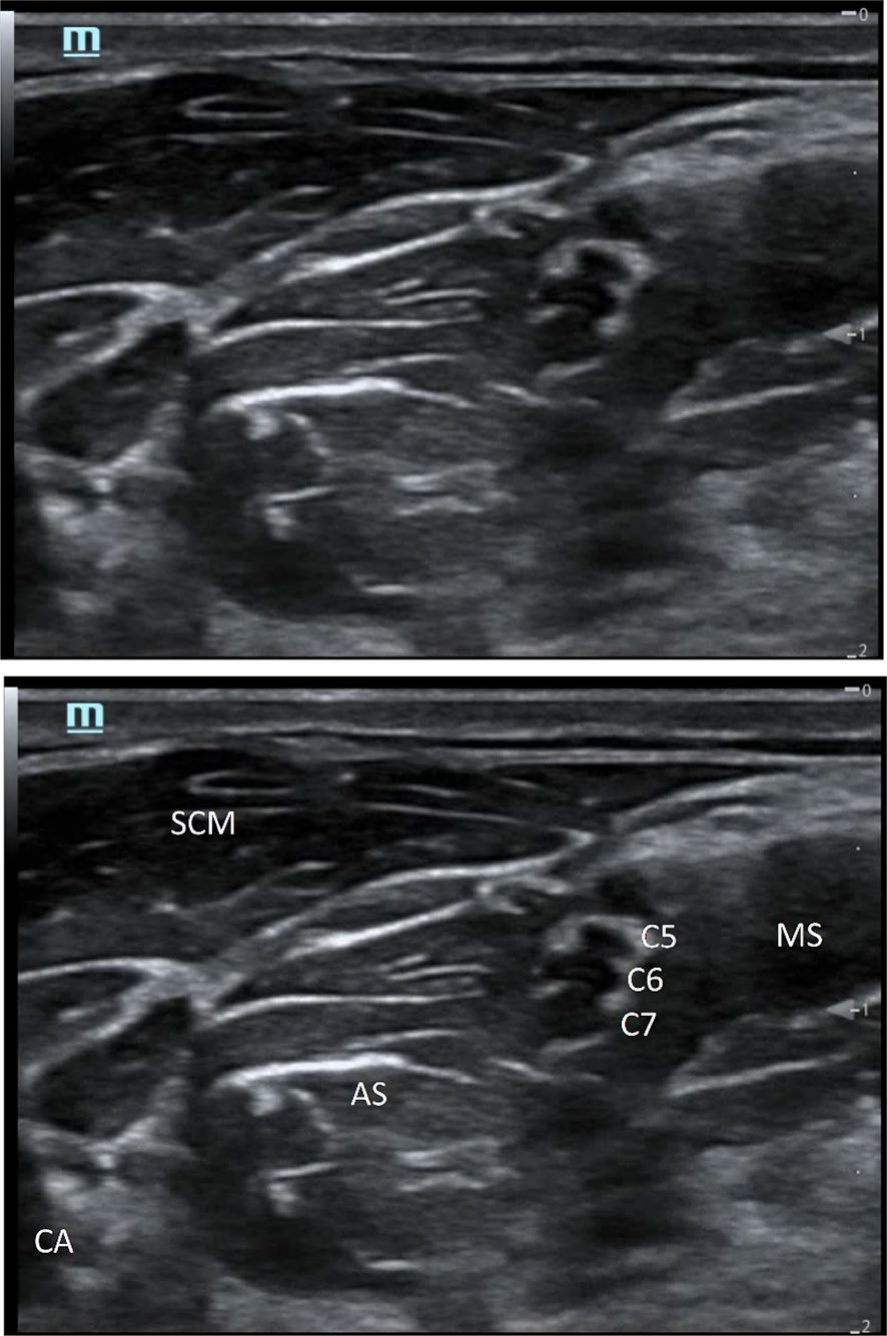
Image acquisition. To pass this assessment, residents needed to acquire an ultrasound image on a healthy volunteer and identify the sternocleidomastoid (SCM), anterior scalene (AS), middle scalene (MS), interscalene groove (C5–7), and carotid artery (CA). They also had to indicate the needle path and site of injection
3. Needle placement assessment: Using an interscalene nerve block trainer with simulated neck anatomy, residents were graded on their ability to guide a needle using in-plane technique into proper position under ultrasound guidance. Scoring was based upon the validated Modified Cheung Checklist (Fig. 3) [24, 25]. Passage was defined as successful placement of the needle at the target site and the presence of fewer than 5 “quality compromising behaviors.”

**Fig. 3:**
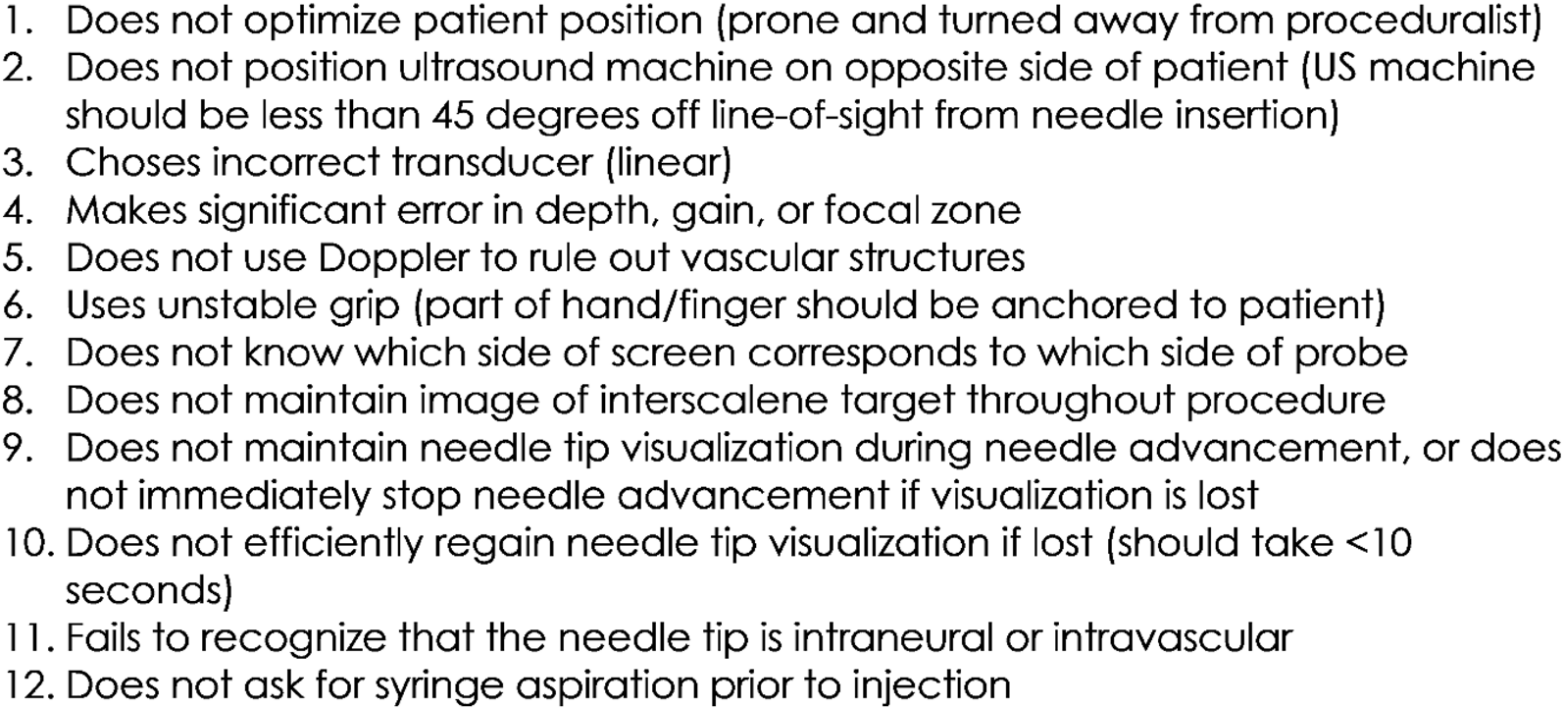
Needle placement scoring metric. Passage of this assessment required identification of the needle tip in the interscalene groove along with the presence of 4 or fewer of the following behaviors (adapted from Wong et al. [25])

Assessments were made by the principal investigator (an ultrasound fellowship trained EP), and a senior EM resident who had undergone training in the ISNB with ultrasound faculty. Assessments were initially performed solely by the PI until an adequate kappa value was calculated for agreement between assessors at which point assessments were variably made by the resident or PI.

The paper, image acquisition, and needle placement assessments were given at the immediate post- and 3 months post-workshop times. Only the paper assessment was given pre-workshop because the practical assessments would hold little value in participants with no prior experience with the ISNB.

For our secondary aim, we performed a retrospective chart review of ED patients receiving the ISNB during 8 months following first workshop compared to the same calendar dates in the year prior. The ED’s electronic medical record was searched for discharge diagnoses of humerus fracture and shoulder dislocation from July 2015 to April 2106 for the “pre-workshop” period and July 2016 to April 2017 for the “post-workshop” period. ICD9 codes were 812 and 831 and ICD10 codes were S42 and S43, respectively (the switch from ICD9 to ICD10 occurred in October 2015). Charts with matching diagnoses were examined for documentation of ISNB administration. Data extraction was performed by a research assistant and included the indication for ISNB, whether procedural sedation was needed after ISNB, and if opiate analgesics were used after ISNB placement.

## Results

Forty-one residents (PGY 1–3) participated in the workshop. Only 3 (7%) reported having previously performed an ISNB. Kappa calculated for inter-rater reliability of the practical assessments showed 100% agreement. Immediately post-workshop, 100% of the participants passed the knowledge and needle placement assessments, and 93% passed the image acquisition assessment. Three months post-workshop, 73% of the residents passed the knowledge assessment, 76% passed the image acquisition assessment, and 100% passed the needle placement assessment (Table 1). Questions’ number 1, 4, 5, and 7 were missed by at least 20% of participants in the 3-month post-workshop assessment. These dealt with block indications, anatomy, anesthetic dosages, and recognition of local anesthetic systemic toxicity, respectively. There were no intraneural or intravascular injections during the needle placement assessments.

**Table 1:** Workshop passage rates

	Pre-workshop	Post-workshop	3 months post-workshop
Paper assessment	9/41 (22%)	41/41 (100%)	30/41 (73%)
Image acquisition	^a^	38/41 (93%)	31/41 (76%)
Needle placement	^a^	41/41 (100%)	41/41 (100%)

^a^Image acquisition and needle placement assessments were not performed pre-workshop due to participants’ lack of exposure to the ISNB at baseline. Passage rate would be anticipated to approach 0%

During the needle placement assessments, there were no intraneural or intravascular injections, and all participants ultimately placed the needle tip at the intended target. In the immediate post-workshop assessment, no resident lost more than 2 points. The most-missed points were for failure to use Doppler and failure to aspirate prior to injecting (27% and 17% of participants, respectively). Three months post-workshop, 2 participants lost 3 points and the remainder lost 2 or fewer. The most-missed points were for failure to use Doppler and failure to always maintain needle tip visualization during needle advancement (34% and 24% of participants, respectively).

In our chart review, there were 2 ISNBs performed in the pre-workshop period and 12 ISNBs performed in the post-workshop period. One was for a proximal humerus fracture and the rest for shoulder dislocation reduction. Pre-workshop, one ISNB was performed by the PI of this study and the other by an EM faculty member. Post-workshop, 6 blocks were performed by residents who attended the workshop, 3 were performed by the PI, and 3 were performed by other EM faculty. Blocks performed by residents were supervised by EM faculty who are not study authors. Lidocaine or bupivacaine was used based on provider preference. There were no immediate complications documented; however, patients were not followed beyond discharge for peripheral nerve injury or other delayed complications. Three patients (21%) required procedural sedation after ISNB, suggesting failed blocks. Two patients (14%) required opiate analgesia after ISNB placement. Ten patients (71%) required neither procedural sedation nor opiate analgesia following ISNB (Table 2).

**Table 2:** Chart review performed over 8 months starting at date of first workshop

	Pre-workshop	Post-workshop
Number of ISNBs	2	12
Complications recorded	0	0
Patients requiring procedural sedation after ISNB	0	3
Patients requiring opiate analgesia after ISNB	0	2

Comparison was same calendar dates in prior academic year. One post-workshop ISNB was for humerus fracture. All others were performed for shoulder reduction

## Discussion

Our study suggests that emergency residents can learn the ISNB, demonstrate competency in practical assessments after a single workshop, and that the ISNB may be an alternative to procedural sedation and opiate use for shoulder dislocation. While residents are particularly adept at ultrasound-guided needle placement, they demonstrated poorest retention of knowledge regarding anatomical landmarks, anesthesia distribution, and early signs of LAST. We recommend that this be used to direct future ISNB instruction and in creation of a bedside reference document that can be reviewed just prior to the procedure.

Regarding our workshop design, we feel that the small group setting (2–4 participants) was an important factor in its success. This allowed for close observation of participants’ performance of procedural skills and quick correction of improper techniques. In addition, much of the instructional energy during workshops was put into foundational knowledge and skills needed for all UGRA applications. While it may seem very labor-intensive for instructors to conduct 15 1-h workshops, the skills learned here greatly simplify future instruction of other nerve blocks. Finally, the overall structure of the workshop—general didactics with image recognition, followed by hands-on image acquisition, and ending with hands-on long-axis needle guidance—is one that we feel can be emulated elsewhere. For others using this workshop design, consideration can be given to pre-workshop study material, as this may enhance knowledge retention. Additional workshops and practice sessions with simulators over the course of residency would also strengthen procedural skills and guard against skill atrophy.

Limitations of the study include lack of assessment of ISNB placement on live patients. While previous studies have suggested that the ability to perform UGRA on a simulator predicts actual performance, it cannot replace real-world experience. Limitations of the chart review include its retrospective nature, single site design, small sample size, and lack of control group. Conclusions about causality cannot be made regarding participation in the workshop and ISNB performance in clinical practice. While no definitive conclusions can be drawn regarding opiate-sparing effects of the ISNB, we do believe that our results are consistent with previous prospective literature demonstrating brachial plexus anesthesia as an alternative to procedural sedation for shoulder reduction. In addition, conclusions regarding the safety of these blocks are limited by lack of patient follow-up beyond discharge. However, the most concerning immediate complications including local anesthetic systemic toxicity, pneumothorax, and respiratory distress from diaphragmatic paralysis were not observed. Finally, this study was performed early on in our group’s collective experience with the ISNB in the ED. We feel that with time and accumulation of experience, ISNB performance and patient outcomes will continue to improve.

## Data Availability

The datasets used in this study are available upon reasonable request from the corresponding author.

## Abbreviations

ISNB: interscalene brachial plexus nerve block
ED: emergency department
EM: emergency medicine
UGRA: ultrasound-guided regional anesthesia
